# Groucho-Mediated Repression May Result from a Histone Deacetylase-Dependent Increase in Nucleosome Density

**DOI:** 10.1371/journal.pone.0010166

**Published:** 2010-04-13

**Authors:** Clint J. Winkler, Alberto Ponce, Albert J. Courey

**Affiliations:** Department of Chemistry and Biochemistry, University of California Los Angeles, Los Angeles, California, United States of America; Duke University, United States of America

## Abstract

Groucho (Gro) is a *Drosophila melanogaster* transcriptional corepressor that directly interacts with the histone deacetylase Rpd3. Although previous studies suggest that this interaction is required for repression of Gro-responsive reporters in cultured cells, the *in vivo* significance of this interaction and the mechanism by which it leads to repression remain largely unexplored. In this study, we show that Gro is partially dependent on Rpd3 for repression, supporting the idea that Rpd3-mediated repression is one mode of Gro-mediated repression. We demonstrate that Gro colocalizes with Rpd3 to the chromatin of a target gene and that this is accompanied by the deacetylation of specific lysines within the N-terminal tails of histones H3 and H4. Gro overexpression leads to wing patterning defects and ectopic repression in the wing disc of transcription directed by the *vestigial* quadrant enhancer. These effects are reversed by the histone deacetylase inhibitors TSA and HC-Toxin and by the reduction of Rpd3 gene dosage. Furthermore, repression of the *vestigial* quadrant enhancer is accompanied by a Gro-mediated increase in nucleosome density, an effect that is reversed by histone deacetylase inhibitors. We propose a model in which Gro-mediated histone deacetylation results in increased nucleosome density leading to transcriptional repression.

## Introduction

The *Drosophila* Groucho (Gro) protein is the founding member of a family of transcriptional corepressors with diverse roles in cell signaling and development. Other members of this family include the human Transducin-like Enhancer of Split (TLE) proteins [1] and the mouse Groucho-related Gene (GRG) proteins [2]. In addition, more distantly related corepressors are found in yeast (e.g., Tup1) [3] and plants [4]. Gro has many roles in *Drosophila* development, including roles in embryonic dorsoventral and terminal patterning, segmentation, sex determination, and wing patterning, while vertebrate Gro orthologs are required for such aspects of vertebrate development as cerebral cortex differentiation and cardiac development [5], [6]. Considering these broad functional roles, it is not surprising that changes in TLE protein expression levels are found in many human cancers including pituitary adenomas [7], [8], lung adenocarcinomas [9], and hematologic malignancies [10].

The role of Gro as a corepressor was initially illuminated through studies of its interaction with the C-terminal WRPW motifs found in bHLH domain-containing transcriptional repressors of the Hairy-Enhancer of split (HES) family [11], [12], [13]. Further studies have shown that Gro is recruited to a variety of target genes by a myriad of DNA-bound repressors. Once recruited to a gene, Gro typically directs long-range repression, i.e., it silences promoters with little regard for the distance between the point of Gro recruitment and the promoter or between the point of Gro recruitment and the enhancers directing activation of the promoter [14]. This is in contrast to the short-range corepressor C-terminal-binding protein (CtBP), which only negates activation by activators bound within a few hundred base pairs of the site to which it is recruited [15], [16]. While Groucho mediates long-range repression, a recent study shows that it can also mediate short-range repression through an interaction with the transcriptional repressor Knirps [17].

Although the mechanism of Gro-mediated long-range repression is unresolved, there are several hints regarding this mechanism. The conserved N-terminal glutamine rich domain of Gro and its mammalian orthologs is predicted to contain two amphipathic helices that could provide an interface for homo-oligomerization through a coiled-coil interaction. Mutations predicted to prevent this interaction inhibit homo-oligomerization and prevent Gro from repressing transcription *in vitro* and *in vivo* [18], [19], [20]. This finding, in combination with the observations that Gro forms high order oligomers and that Gro binds to deacetylated histones suggests that the movement of Gro, perhaps through spreading along chromatin, is required for long-range repression [21], [22].

Additional observations suggest that Gro may repress transcription by changing chromatin structure. First, Groucho family proteins directly interact with the *Drosophila* histone deacetylase Rpd3 or its mammalian ortholog HDAC1, and this interaction plays a functional role in the repression of target genes in cultured cells and *Drosophila* embryos [23], [24], [25]. Second, Grg3, a mammalian Groucho family protein, is able to condense and aggregate reconstituted nucleosomal arrays *in vitro* via an interaction with the tails of histones H3 and H4 [26]. Third, recent ChIP studies show colocalization of Rpd3 with Gro in the long-range repression of a *lacZ* reporter gene in the *Drosophila* embryo [27]. These findings suggest a repression model in which recruitment of Rpd3 by Gro leads to the organization of chromatin into a condensed and repressed state by removal of acetyl groups from histone tails. The observation that Gro binds to hypoacetylated histone tails suggests that this repressed state may be re-enforced by the recruitment of additional Gro to hypoacetylated chromatin [22].

In this study, we further characterize the connection between histone deacetylation and Gro-mediated repression. We show that Gro is partially dependent on Rpd3 for repression in cultured cells and that this interaction results in the deacetylation of specific lysines in histones H3 and H4. To extend these findings to the intact organism, we carried out experiments showing that histone deacetylase inhibitors or reduction of *Rdp3* gene dosage significantly reduce the defects resulting from overexpression of Gro in the developing *Drosophila* wing. Furthermore, the histone deacetylase inhibitors were found to interfere with Gro-dependent repression in the wing disc via the *vestigial* quadrant enhancer, a known Gro regulatory target. In addition, recruitment of Gro to the chromatin increases nucleosome density and this increase is blocked by a histone deacetylase inhibitor. Significantly, this increase in nucleosome density is not dependent upon a change in transcriptional activity suggesting that it is not merely a consequence of decreased transcription, but may lead to repression. Thus, Gro may repress transcription, at least in part, by recruiting histone deacetylases. The resulting Groucho-mediated decrease in histone acetylation levels may lead to increased nucleosome density and/or stability and therefore to transcriptional repression.

## Results

### Gro-mediated repression in cultured cells is partially dependent on Rpd3

Previous studies have shown that the histone deacetylase inhibitor Trichostatin A (TSA) dramatically reduces the ability of Gro to repress a reporter gene in cell culture when directly targeted to the promoter by fusion to the Gal4 DNA binding domain [23], [25]. Additionally, the glycine proline rich (GP) domain of Gro, which has been shown to interact directly with Rpd3, functions as a repression domain, the activity of which is enhanced by Rpd3 overexpression, when targeted to the same reporter gene [23]. These studies suggest that Gro relies on histone deacetylase function, at least in part, for repression.

**Figure 1:**
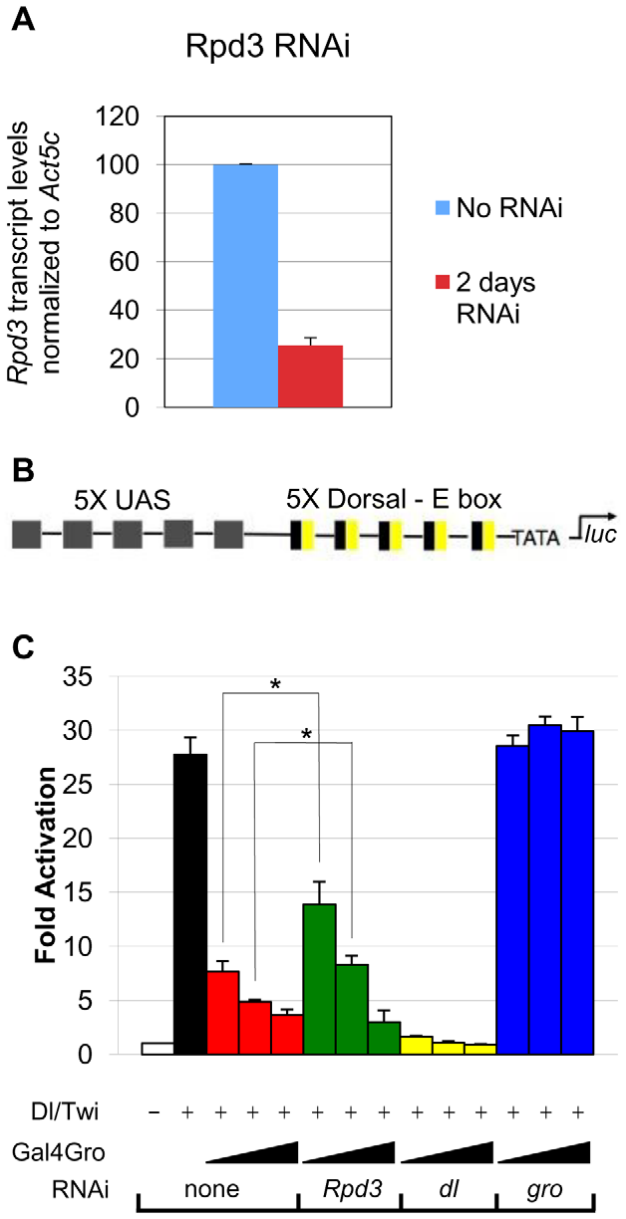
Gro-mediated repression is partially dependent on Rpd3. (**A**) RNAi knockdown of *Rpd3* leads to a 75% reduction in transcript levels. Transcript levels were measured by quantitative RT-PCR. (**B**) The *luciferase* reporter construct used in the repression assay. The reporter contains five copies of the yeast upstream activation sequence (UAS), which binds directly to a Gal4-Gro fusion protein containing the DNA binding domain of the yeast activator Gal4. Additionally, the reporter contains five copies of the Dorsal – E box enhancer, which directs synergistic gene activation by the DNA binding activators Dorsal and Twist. (**C**) Basal reporter activity (white bar); Synergistic activation of the reporter by cotransfection of *dl* and *twi* (black bar); Cotransfection of 30, 100 & 300 ng of vector encoding Gal4-Gro leads to repression of the reporter in a dose dependent manner (red bars); RNAi knockdown of *Rpd3* results in inhibition of Gro-mediated repression in the presence of 30 and 100 ng, but not 300 ng of vector encoding Gal4-Gro (green bars); RNAi knockdown of *dl* results in loss of synergistic activation (yellow bars); RNAi knockdown of *gro* results in a loss of repression (blue bars). Bars indicate average (±S.E.) of three to four independent biological replicates. A single asterisk (*) indicates indicate pairwise comparisons for which p<0.05. A double asterisk (**) indicates pairwise comparisons for which p<0.01.

To further assess the dependence of Gro on histone deacetylation, we used RNA interference to knock down Rpd3 in *Drosophila* S2 cells (Fig. 1A). The *luciferase* reporter employed in these experiments contains an artificial enhancer consisting of multimerized binding sites for the Dorsal and Twist activators (DL-Ebox) as well as multimerized binding sites for Gal4 (5X-UAS) (Fig. 1B). As previously shown, the combination of Dorsal and Twist strongly activates the reporter (Fig. 1C, black bar), while the addition of Gal4-Gro results in strong dose-dependent repression (Fig. 1C, red bars). Knocking down Dorsal expression levels by RNAi leads to a significant reduction in activation (Fig. 1C, yellow bars), while knocking down Gro expression by RNAi leads to loss of repression of the reporter (Fig. 1C, blue bars).

We investigated the role of Rpd3 in repression as a function of Gal4-Gro concentration. At low to intermediate levels of Gal4-Gro, Rpd3 knockdown leads to a partial loss of repression by Gro (Fig. 1C, green bars, compare to red bars). At high levels of Gal4-Gro, however, Rpd3 knockdown had no influence on repression. These findings suggest that Gro employs both Rpd3-dependent and Rpd3-independent modes of repression. This is consistent with previous studies in *Rpd3* mutant *Drosophila* embryos suggesting that some but not all Gro targets are derepressed in the absence of Rpd3 [24]. Multiple modes of repression could allow Gro to fine tune gene activity in response to promoter context and cellular environment.

### Gro-mediated repression correlates with Rpd3 recruitment and changes in histone acetylation patterns

**Figure 2:**
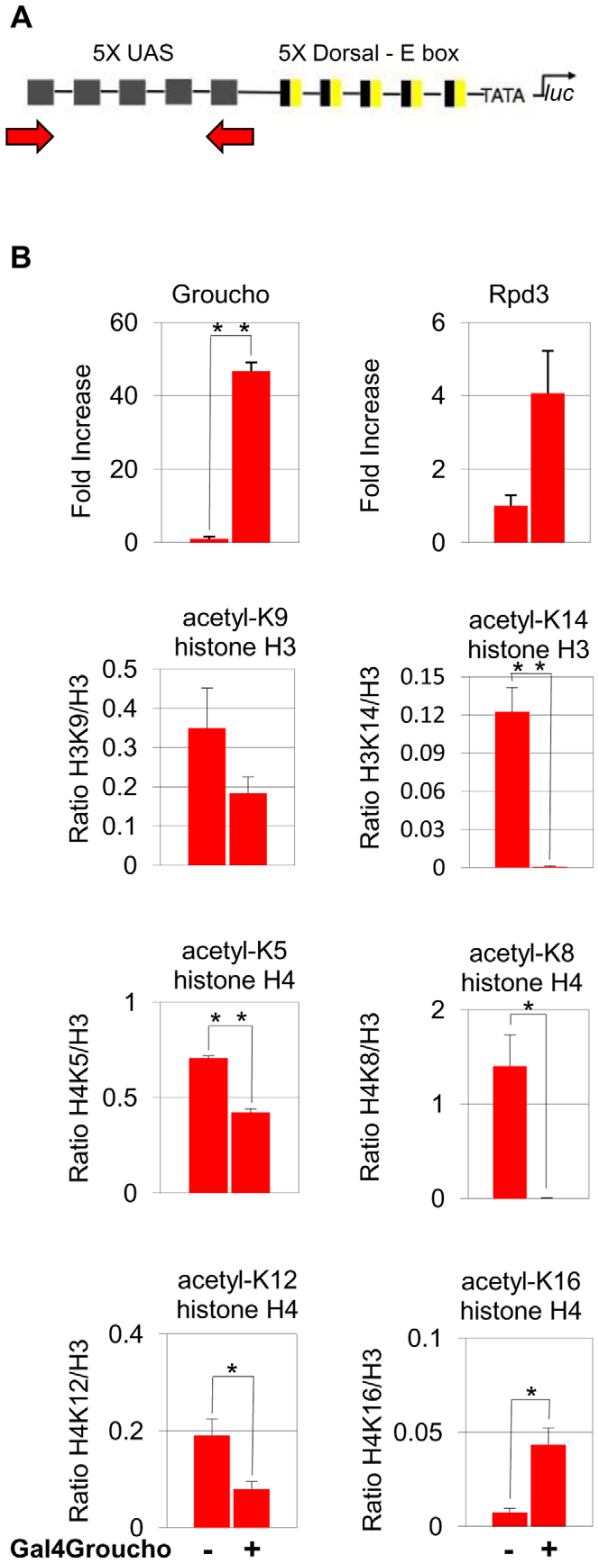
Gro-mediated repression correlates with Rpd3 recruitment and changes in histone acetylation patterns. (**A**) The *luciferase* reporter construct used in ChIP assays. The reporter contains five copies of the yeast upstream activation sequence (UAS), which can directly bind Gal4-Gro. The arrows show the position of the amplicon, which is centered over the UAS. (**B**) Copper induction of *Gal4-Gro* expression results in a 45-fold increase in the Gro ChIP signal and a 4 fold increase in Rpd3 ChIP signal at the UAS 48 hours after induction. Gro and Rpd3 ChIP data are plotted as fold increase. This is derived by dividing both the induced and non-induced values (as % input) by the non-induced value. A decrease in acetylation of lysines 9 and 14 on histone H3 is observed at the site of Gal4-Gro binding. A decrease in acetylation of lysines 5, 8, and 12 on histone H4 is also observed at the site of Gal4-Gro binding. An increase in acetylation of histone H4 lysine 16 is observed. ChIP signals (as % input) from antibodies recognizing acetylated forms of histones were normalized to total histone H3 levels using an antibody that recognizes an area of histone H3 that is not post-translationally modified. Specifically, the acetylated histone ChIP signal was divided by the non-translationally modified histone ChIP signal. Bars indicate average (±S.E.) of three independent biological replicates. A single asterisk (*) indicates indicate pairwise comparisons for which p<0.05. A double asterisk (**) indicates pairwise comparisons for which p<0.01. The p-values for the Rpd3 and H3K9 data (0.060 and 0.058, respectively) slightly exceed our cutoff (p<0.05) for statistical significance.

To further elucidate the relationship between Gro and Rpd3 during Gro-mediated repression in S2 cells, we carried out chromatin immunoprecipitation (ChIP) experiments to look at specific changes in lysine acetylation. These experiments were performed in a cell line containing a stably integrated copper inducible transgene encoding the Gal4-Gro fusion protein. The cells also contain an integrated *luciferase* reporter gene with five Gal4 binding sites upstream of the promoter (Fig. 2A). After 48 hours of copper induction, we observed a >45-fold increase in the level of Gal4-Gro and a 4-fold increase in the level of Rpd3 associated with the Gal4 binding sites. We also observe significant deacetylation of histone H3 K9 and K14 and histone H4 K5, K8, and K12. Interestingly, we also observe increased acetylation of histone H4 K16 (Fig. 2B). This observation is consistent with studies of H4 K16 acetylation patterns in yeast (see discussion).

### Histone deacetylase inhibitors prevent wing patterning defects resulting from Gro overexpression

Gro has multiple roles in wing development including roles in the Notch, Wingless, and Dpp pathways [28], [29], [30]. The level of Gro appears to be critical for normal patterning of the wing as overexpression of Gro in the wing disc leads to a variety of wing defects including wing vein patterning defects and wing blistering. The vein patterning defects may result from the role of Gro in mediating repression of pro-vein genes by E(spl), a target of the Notch signal, while the blistering may result from improperly coordinated growth of the dorsal and ventral epithelia that comprise the wing. To assess the role of histone deacetylation in Gro mediated repression, we therefore examined the effect of histone deacetylase inhibitors on the wing patterning defects resulting from overexpression of Gro directed by a *UAS-Gro* transgene. Histone deacetylase inhibitors do not, on their own, result in wing patterning defects at the concentrations used in these studies (data not shown).

**Figure 3:**
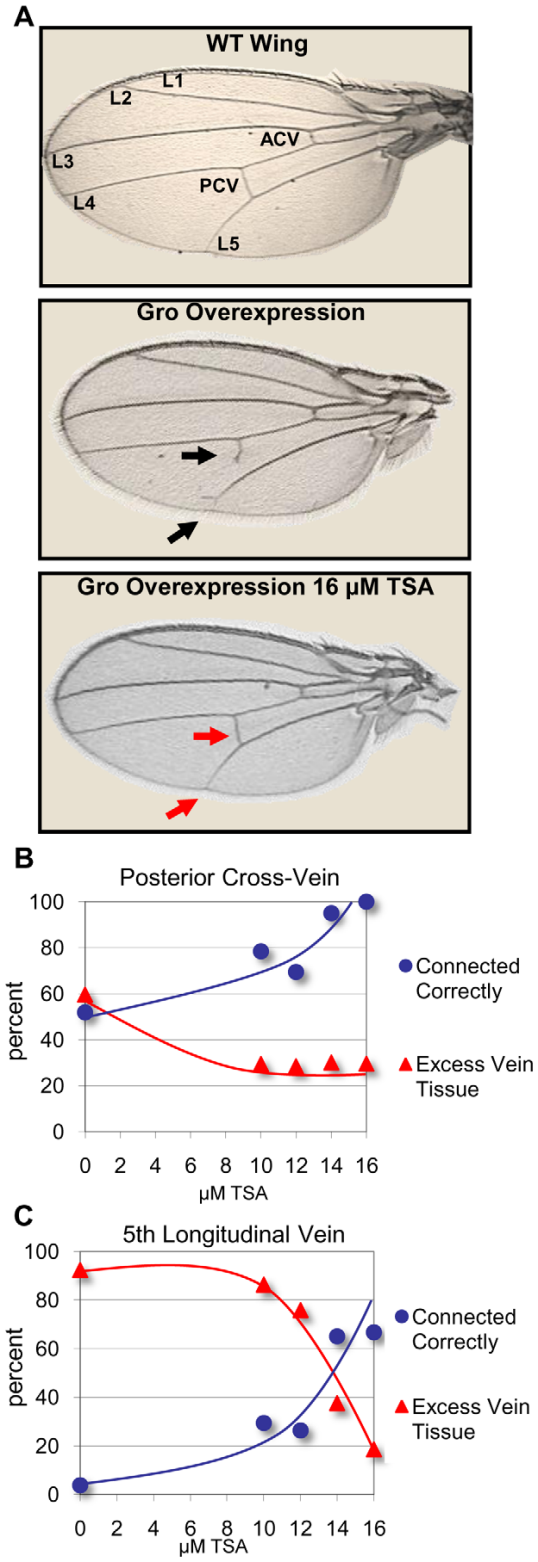
Trichostatin A prevents wing vein patterning defects resulting from moderate overexpression of Gro in the wing blade. (**A**) A wildtype *Drosophila* wing showing the five main longitudinal veins (L1-5), the anterior crossvein (ACV) and the posterior crossvein (PCV) (top panel). Overexpression of Gro by the 3^rd^ instar wing disc driver *C765-Gal4* at 25°C leads to adult wing vein patterning defects (middle panel). The PCV fails to connect the L4 to the L5, and the L5 fails to connect to the wing margin. Additionally, both veins show ectopic improperly directed growth (black arrows). Adult wings derived from larvae raised in food containing 16 µM TSA show significant rescue of the vein patterning defects resulting from Gro overexpression (bottom panel, red arrows). (**B**) Adult wings derived from larvae overexpressing Gro (driven by *C765-Gal4*) that are raised in increasing concentrations of TSA show a dose dependent increase in correctly connected PCV and a decrease in excess PCV tissue. (**C**) Adult wings from larvae overexpressing Gro (driven by *C765-Gal4*) that are raised in food containing increasing concentrations of TSA show a dose dependent increase in correctly connected L5 and a decrease in excess L5 tissue. The following numbers of wings were examined: DMSO alone (n = 52), 10 µM TSA (n = 51), 12 µM TSA (n = 95), 14 µM TSA (n = 40), 16 µM TSA (n = 27).

Moderate Gro overexpression throughout the wing pouch (directed by the *C765-Gal4* driver) results in a posterior cross-vein (PCV) that fails to connect the 4^th^ and 5^th^ longitudinal veins (L4 and L5), and an L5 vein that fails to connect to the wing periphery (Fig. 3A). In each case, the failure to connect is often accompanied by excess incorrectly oriented vein growth. The PCV and L5 vein patterning defects were examined in flies grown on control food containing the carrier DMSO and on food containing concentrations of TSA ranging from 10 to 16 µM. For the PCV and the L5, as the concentration of TSA increases, the percentage of correctly connected veins increases and the percentage of excess vein tissue decreases (Figs. 3B and 3C, Table S2).

**Figure 4:**
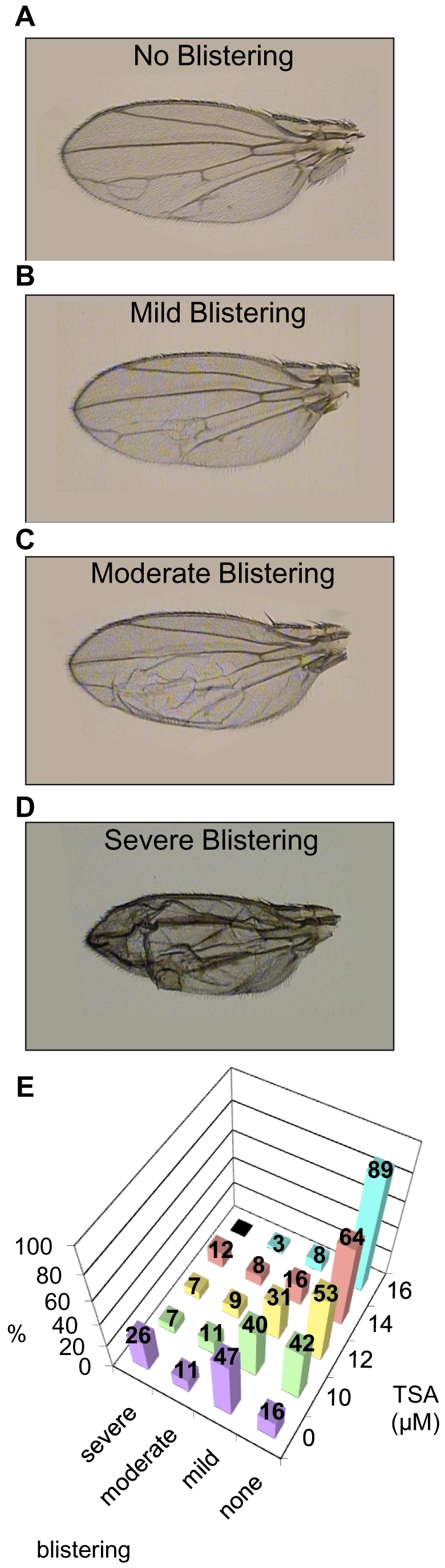
Trichostatin A prevents wing blistering defects resulting from high level overexpression in the wing blade. Overexpression of Gro in the female 3^rd^ instar wing disc directed by the *MS1096-Gal4* driver at 25°C leads to adult wing blistering. The severity of blistering varies and is categorized as (**A**) no blistering, (**B**) mild blistering (≤25% of the wing affected), (**C**) moderate blistering (between 25 and 50% of the wing affected) and (**D**) severe blistering (> 50% of the wing affected). (**E**) Adult wings derived from larvae overexpressing Gro (driven by *MS1096-Gal4* at 25°C) that are raised in food containing increasing concentrations of TSA show a dose dependent shift of blistering severity, moving toward a less severe phenotype as the concentration of TSA increases. The following numbers of wings were examined at the given TSA concentrations: DMSO alone (n = 89), 10 µM (n = 45), 12 µM (n = 45), 14 µM (n = 25), 16 µM (n = 62).

High level overexpression of Gro in the dorsal wing pouch (directed by the *MS1096-Gal4* driver) leads to wing blistering, likely due to improper adhesion between the dorsal and ventral epithelia that comprise the wing blade [31]. To allow quantitative analysis of this phenotype we scored wings as severely blistered (Fig. 4D), moderately blistered (Fig. 4C), mildly blistered (Fig. 4B), or unblistered (Fig. 4A). In the absence of TSA, wings overexpressing Gro driven by the *MS1096-Gal4* driver show 26% severe blistering, 11% moderate blistering, 47% mild blistering and 16% no blistering (Fig. 4E, purple bars). With increasing concentrations of TSA in the food during larval development, we observed decreasing severity of the wing blistering. At the highest concentration of TSA used in this experiment (16 µM), 89% of the wings exhibited no blistering (Fig. 4E, blue bars).

In addition, to carrying out studies with TSA, we carried out similar studies using HC-Toxin. While TSA inhibits both Class I and Class II histone deacetylases, HC-toxin is thought to be specific for the Class I enzymes [32]. Similarly to TSA, HC-toxin reduced the severity of wing defects (Fig. S1, Table S1). In conclusion, two different histone deacetylase inhibitors rescue the developmental defects induced by Gro overexpression, strongly suggesting that full Gro activity in development is at least partially dependent on histone deacetylase function.

### Reducing *Rpd3* gene dosage rescues wing patterning defects resulting from Gro overexpression

**Figure 5:**
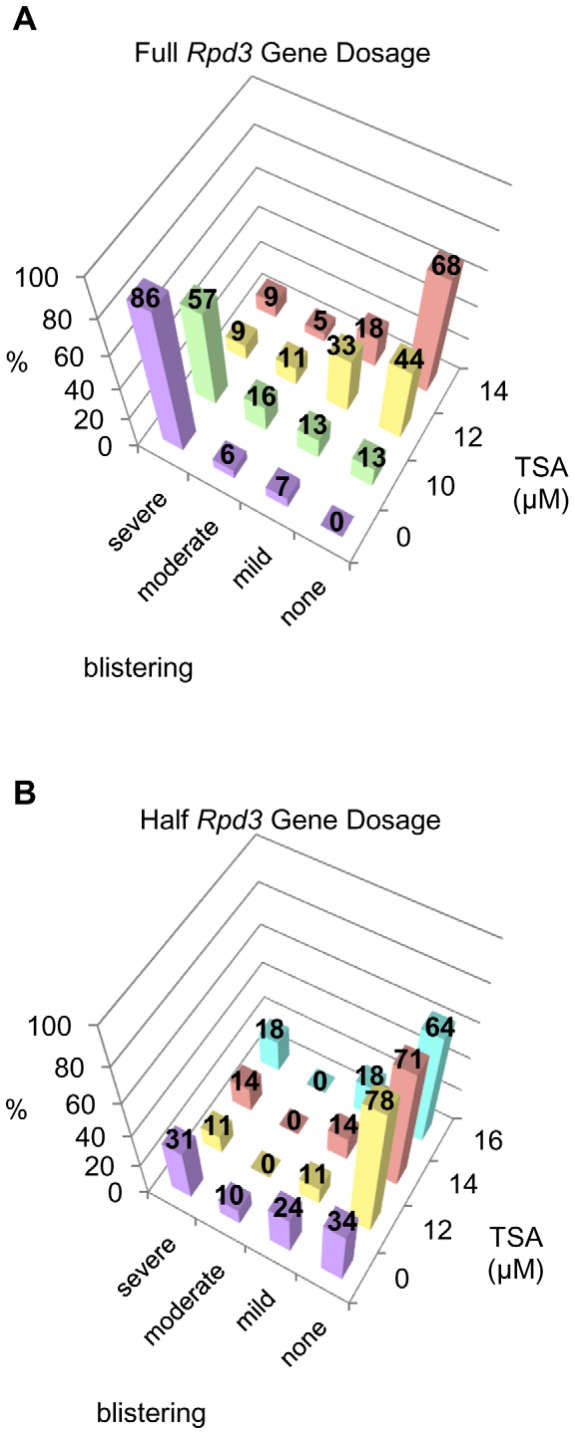
Reduction of *Rpd3* gene dosage rescues wing patterning defects resulting from Gro overexpression. Overexpression of Gro by the 3^rd^ instar wing disc driver *Serrate-Gal4* at 25°C leads to adult wing blistering. The severity of blistering is categorized as described in the legend to Fig. 4. (**A**) Adult wings derived from larvae overexpressing Gro (driven by the *Serrate-Gal4* driver at 25°C) in the presence of two functional copies of the *Rpd3* gene show a monotonic dose dependent decrease in wing blistering severity as TSA concentration is increased. The following numbers of wings were examined at the given TSA concentrations: DMSO alone (n = 170), 10 µM (n = 61), 12 µM (n = 55), 14 µM (n = 22). (**B**) Adult wings derived from larvae overexpressing Gro (driven by the *Serrate-Gal4* driver at 25°C) in the presence of one functional copy of the *Rpd3* gene show a dramatic decrease in blistering severity at 12 µM TSA. However, as the TSA concentration is raised to 14 and 16 µM, the trend reverses and blistering becomes more severe. The following numbers of wings were examined at the given TSA concentrations: DMSO alone (n = 87), 12 µM (n = 18), 14 µM (n = 7), 16 µM (n = 11).

If HDAC inhibitors rescue Gro overexpression phenotypes by reducing the ectopic repression of target genes through decreased levels of Rpd3 function, one would expect a decrease in *Rpd3* gene dosage to have the same effect. To test this hypothesis, we used a mutant allele of *Rpd3* created by imprecise excision of the P-element in the hypomorphic *Rpd3* ^04556^ allele. Southern blot and PCR analysis show that this allele (*Rpd3^Δ^*) contains an ∼2000 bp deletion that spans the 1^st^ and 2^nd^ exons, and a portion of the 3^rd^ exon, and we presume it to be a null allele (data not shown). It is recessive larval lethal. To determine the role of Rpd3 in Gro function, we compared the effect of Gro overexpression in the wild-type wing to the effect of Gro overexpression in *Rpd3^Δ^/+* heterozygous wings. *Rpd3^Δ/+^* wings show no developmental defects resulting from decreased *Rpd3* levels alone. In this experiment, high levels of Gro expression (driven by *Serrate-Gal4*) in the presence of two functional copies of *Rpd3* led to extensive wing blistering – 86% of the wings showed severe blistering, 6% moderate blistering, 7% mild blistering, and 0% no blistering (Fig. 5A, purple bars). In the presence of just one copy of wild-type *Rpd3*, the average severity of the phenotype was much reduced – 31% showed severe blistering, 10% moderate blistering, 24% mild blistering, and 34% no blistering (Fig. 5B, purple bars).

As might be expected, lower concentrations of TSA are required to alleviate the Gro overexpression phenotype in *Rpd3^Δ/+^* heterozygous wings than in *Rpd3^+/+^* wings. In heterozygotes, the fraction of unblistered wings is already maximal at 12 µM TSA, the lowest concentration tested, and decreases slightly at higher TSA concentrations, presumably because a minimum level of Rpd3 activity is required for normal development (Fig. 5B). In *Rpd3* ^+/+^ animals by contrast, the fraction of unblistered wings increases monotonically as the TSA concentration is increased to 14 µM (Fig. 5A).

### Trichostatin A (TSA) and HC-Toxin inhibit Gro-dependent repression via the *vestigial* quadrant enhancer

**Figure 6:**
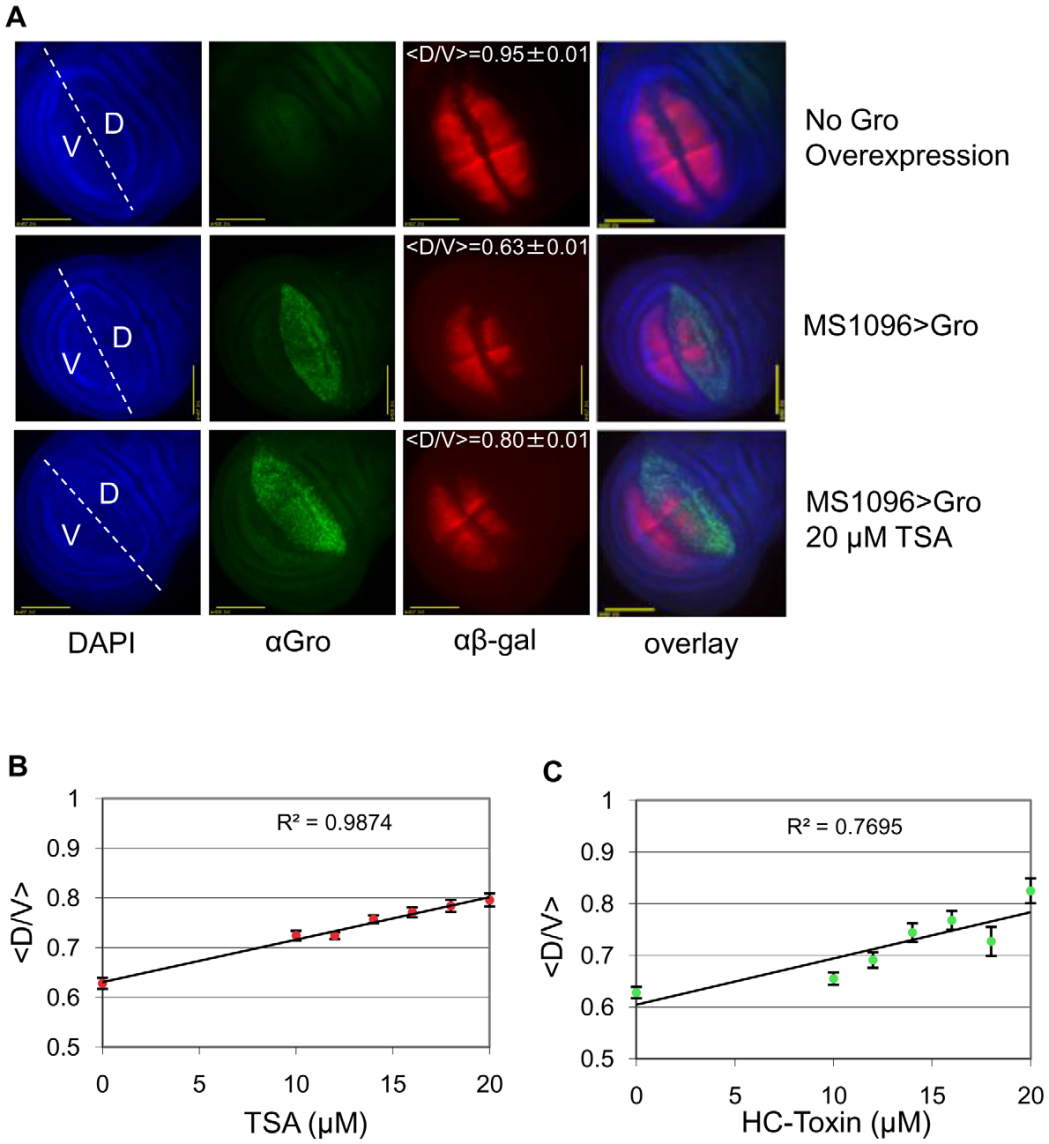
TSA and HC-Toxin inhibit Gro dependent repression via the *vestigial* quadrant enhancer. (**A**) DAPI staining showing all nuclei of the 3^rd^ instar wing disc, Gro staining showing dorsal wing pouch overexpression (driven by *MS1096-Gal4* in females), and β-gal staining showing expression of the *vestigial* quadrant enhancer that is bisected by the dorsoventral boundary. **Top row:** β-gal staining showing *vgQ-lacZ* transgene expression in a 3^rd^ instar wing disc under wildtype conditions. **Middle row:** β-gal staining showing a reduction of *vgQ-lacZ* expression in the dorsal half of the wing pouch where it colocalizes with Gro overexpression **Bottom row:** β-gal staining showing an expanded *vgQ-lacZ* expression in the presence of Gro overexpression when raised on food containing 20 µM TSA. <D/V> is the average value (± standard deviation) for the length of the dorsal stripe divided by the length of the ventral stripe of *vgQ-lacZ* expression. (**B**) <D/V> as a function of TSA concentration. Increasing TSA concentration results in a dose dependent decrease in Gro-mediated repression of the *vgQ-lacZ* transgene. The p-value for the slope of the line is less than 0.01. The following numbers of wings were examined at the given TSA concentrations: DMSO alone (n = 71), 10 µM (n = 46), 12 µM (n = 59), 14 µM (n = 48), 16 µM (n = 35), 18 µM (n = 31), 20 µM (n = 15). (**C**) <D/V> ratio as a function of HC-Toxin concentration. Increasing HC-Toxin concentration also results in a dose dependent decrease in Gro-mediated repression on the *vgQ-lacZ* transgene. The p-value for the slope of the line is less than 0.01. The following number of wings were examined at the given HC-Toxin concentrations: DMSO alone (n = 71), 10 µM (n = 60), 12 µM (n = 27), 14 µM (n = 18), 16 µM (n = 14), 18 µM (n = 5), 20 µM (n = 6).

The quadrant enhancer of the *vestigial* gene (*vgQ*) is a Dpp-responsive enhancer responsible for driving expression of the gene in a symmetrical domain around the intersection of the anteroposterior and dorsoventral boundaries in the 3^rd^ instar wing disc [33], as revealed by anti-β-gal staining of wing discs containing a *vgQ-lacZ* reporter (Fig. 6A). Brinker, a repressor that antagonizes Dpp signaling, binds the *vgQ* enhancer and recruits Gro for repression of the transcription directed by this enhancer. Brinker is localized in a gradient with high concentrations at the anterior and posterior margins and low concentrations at the anteroposterior midline. As a result, Brinker working with Gro serves to delimit the anteroposterior extent of the *vestigial* expression domain [19], [30].

When Gro overexpression is driven in just the dorsal half of the wing pouch, the result is a reduction in the width of the dorsal half of the *vgQ* expression domain (Fig. 6A, compare top and middle rows). Consistent with previous observations of wing disc target genes where Brinker recruits Gro as a corepressor [19], this narrowing of the width of the domain suggests that Brinker function is sensitive to the absolute level of Gro and that, at higher concentrations of Gro, lower concentrations of Brinker are sufficient for repression. To quantify the level of repression resulting from overexpression of Gro in the dorsal compartment, we determined the ratio of the width of the *vgQ*-lacZ expression domain in the dorsal compartment to the width of the expression domain in the ventral compartment. We term this value the *vgQ* D/V ratio. The average *vgQ* D/V ratio (<D/V>) in the absence of Gro overexpression is 0.95, while dorsal compartment specific Gro overexpression decreases the ratio to 0.63 (Figs. 6A, top and middle rows). When Gro is overexpressed in the presence of 20 µM TSA, <D/V> is 0.80 (Fig. 6A, bottom row) signifying a decrease in Gro-mediated repression in the presence of the inhibitor. When <D/V> is measured as a function of TSA or HC-toxin concentration we observe roughly linear trends (Fig. 6B and C). The slopes for both lines have p-values less than 0.01 indicating that the correlation between TSA concentration and D/V ratio is highly statistically significant. Thus, Gro-mediated repression in a developmental setting is sensitive to the level of histone deacetylase activity.

### TSA prevents a Gro-mediated increase in nucleosome density at the *vestigial* quadrant enhancer

**Figure 7:**
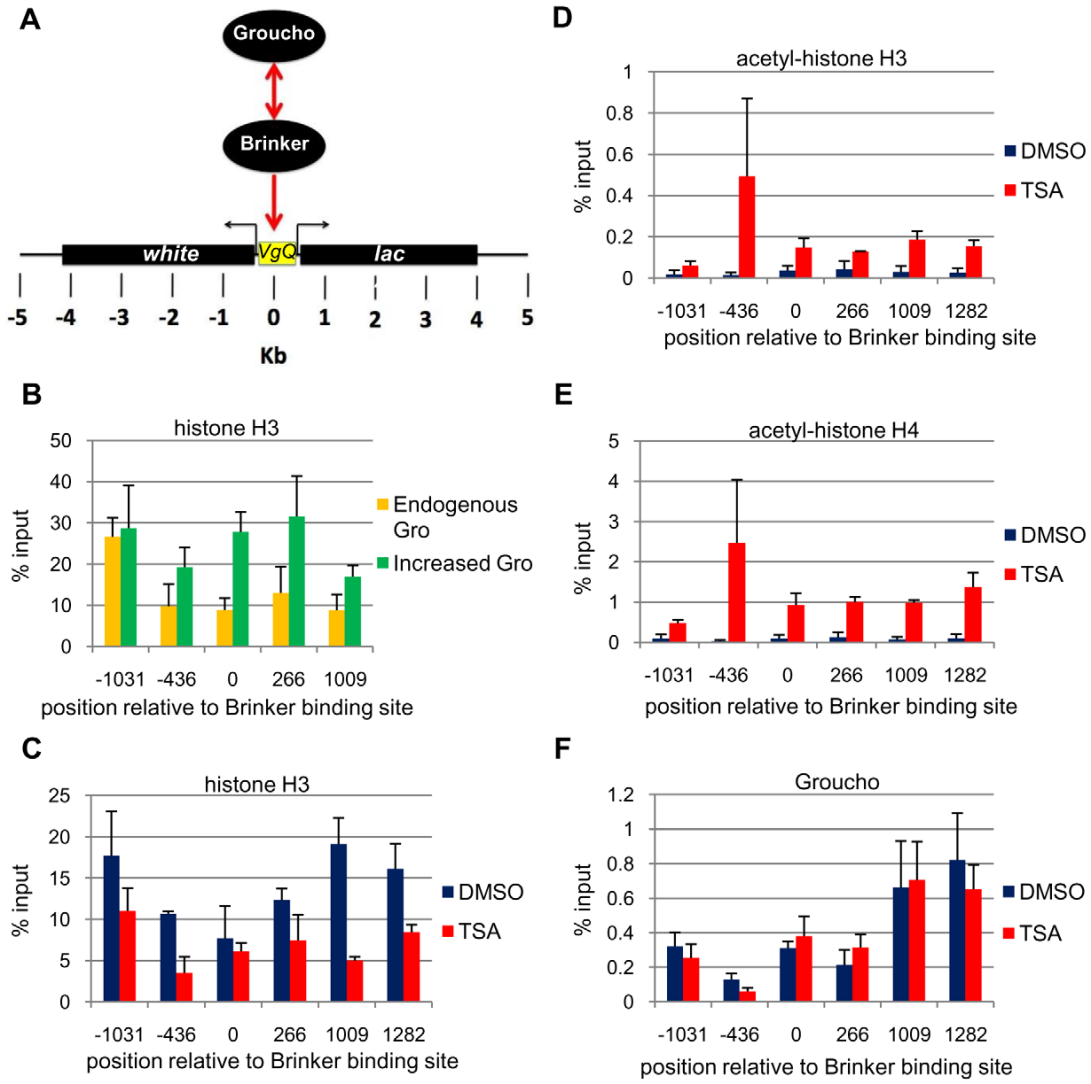
TSA prevents a Gro-mediated increase in nucleosome density within the *vestigial* quadrant enhancer. (**A**) The *vgQ-lacZ* transgene contains the *vestigial* quadrant enhancer, which is flanked by the *Drosophila white* gene on the left and the *lacZ* reporter gene on the right. Brinker directly binds the quadrant enhancer and recruits Gro for repression. (**B**) Overexpression of Gro with the *MS1096-Gal4* driver in males at 29°C results in a significant increase in wing disc histone H3 ChIP signal (green bars) in areas flanking the Gro recruitment site in the *vgQ-lacZ* transgene when compared to flies not overexpressing Gro (orange bars). Bars indicate average (±S.E.) of three independent biological replicates. (**C**) TSA treatment (red bars) results in a significant decrease in wing disc histone H3 ChIP in areas flanking the Gro recruitment site in the *vgQ-lacZ* transgene compared to wing discs from flies raised in the absence of TSA (blue bars). Bars indicate average (±S.E.) of two independent biological replicates. (**D**) An antibody that recognizes acetylated lysines 9 and 14 on histone H3 yields increased wing disc ChIP signal throughout the transgene in larvae raised in 16 µM TSA (red bars) compared to larvae raised in the absence of TSA (blue bars). Bars indicate average (±S.E.) of two independent biological replicates. (**E**) An antibody that recognizes acetylated lysines 5, 8, 12 and 16 on histone H4 yields increased wing disc ChIP signal throughout the transgene in larvae raised in 16 µM TSA (red bars) compared to larvae raised in the absence of TSA (blue bars). Bars indicate average (±S.E.) of two independent biological replicates. (**F**) Binding of Gro to the reporter shows no significant difference in the presence (red bars) or absence (blue bars) of TSA. Bars indicate average (±S.E.) of three independent biological replicates.

The above results show that repression by Gro via the *vgQ* enhancer is reduced under conditions of decreased histone deacetylase activity, thus suggesting that Gro may direct a change in the chromatin environment. We addressed this through ChIP assays on the *vgQ-lacZ* transgene (Fig. 7A) with an antibody that recognizes a portion of histone H3 that is not post-translationally modified. Overexpression of Gro in the dorsal wing pouch results in an increase in the anti-histone H3 ChIP signal across the *vgQ-lacZ* reporter (Fig. 7B). The average increase in histone H3 density is 1.9-fold across the five amplicons spanning the region from −1031 to +1009 (p<0.01). Thus, Gro-mediated repression correlates with a significant increase in nucleosome density.

Previous studies suggest that active genes often exhibit decreased nucleosome density relative to inactive genes [34], [35]. This could be due, for example, to the disruption of chromatin structure by actively elongating RNA polymerase [36]. Thus, the increase in nucleosome density resulting from Gro overexpression could be a consequence rather than a cause of transcriptional repression. We believe this is unlikely because the change in the expression domain of the *vgQ* reporter upon overexpression of Gro is modest (see Fig. 6, for example) compared to the greater than two-fold increase in the histone H3 ChIP signal observed at most sites in the reporter. However, to further test the possibility that the change in nucleosome density results from a change in the transcriptional state of the reporter, we carried out H3 ChIP in eye discs, a tissue in which the *vgQ* reporter is not active (data not shown) [37]. Similar to what we observe in wing discs, where the reporter is transcribed, we observe an average increase of 2.6-fold in histone H3 density across the reporter in eye discs (p<0.05) (Fig. S2).

We next examined the effect of TSA on nucleosome density and acetylation state. Histone H3 ChIP revealed an average decrease in nucleosome density of 2-fold over the *vgQ-lacZ* transgene in the presence of TSA (p<0.001) (Fig. 7C). The greater nucleosome density in the absence of TSA is seen in the regions flanking the *vgQ* enhancer, but not in the *vgQ* enhancer itself, possibly because sequence specific factors binding to the enhancer disrupt nucleosome assembly. Immunoprecipitation with antibodies raised against multiply acetylated forms of the histones H3 and H4 tails reveals that TSA leads to increased acetylation of histone H3 (Fig. 7D) and histone H4 (Fig. 7E).

Gro interacts with hypoacetylated histones, suggesting the possibility that it uses deacetylated histones as a platform to spread across a gene during repression. To address this possibility, we carried out Gro ChIP on wing discs from TSA treated and untreated flies. We find that TSA does not influence Gro recruitment to the *vgQ* enhancer (Fig. 7F). This finding was surprising based on previously published interactions between Gro and histone tails and suggests the interaction between Gro and the target gene consists of more than a binary association with hypoacetylated histone tails.

## Discussion

The findings presented here suggest that Gro works by a histone deacetylase-dependent mechanism. Our cell culture studies suggest that Gro recruits *Rpd3* leading to the deacetylation of the N-terminal tails of histones H3 and H4. Through the use of histone deacetylase inhibitors and manipulation of the *Rpd3* gene dosage, we have also shown that histone deacetylase contributes to Gro activity in an intact tissue, namely the *Drosophila* wing disc. Furthermore, our findings suggest that Gro-mediated histone deacetylation leads to increased nucleosome density, which results in a repressed state.

### Histone deacetylase dependent mechanisms of Gro-mediated repression in cultured cells

Our results show that Gro requires Rpd3 function for full repression activity in cultured cells. We also found that Gro-mediated repression in cell culture is accompanied by an increase in chromatin-associated Rpd3, and reduction in the acetylation of five of six lysines in the N-terminals tails of histones H3 and H4. These findings support previous studies suggesting that Gro modulates the expression of target genes, at least in part, by directing deacetylation of histone tails [25], [27].

Interestingly, Gro-mediated repression in S2 cells is accompanied by a marked increase in the acetylation of histone H4, lysine 16 (H4 K16). While the meaning of this observation is unclear, it is consistent with a study showing that H4 K16 acetylation in yeast exhibits a marked anticorrelation with actively transcribed genes [38]. It is possible that Gro directly recruits an H4 K16 specific histone acetyltransferase or that the hyperacetylation of H4 K16 is an indirect consequence of deacetylation of other lysines in the histone tails. We note that, in some circumstances, H4 K16 positively correlates with transcriptional activity [39], [40]. Thus, the consequences of H4 K16 acetylation appear to be context dependent.

Our cell culture studies are consistent with previous studies that suggest additional mechanisms of Gro-mediated repression. At low Gro concentration, Rpd3 knockdown leads to derepression, while at high Gro concentration, no derepression is observed. This is consistent with genetic studies showing embryonic patterning defects to be much less severe in *Rpd3* mutants than in *gro* mutants [24]. Considering the myriad of repressors that utilize Gro to repress genes in response to a diverse array of signals, it is perhaps not surprising that Gro should employ multiple modes to repress transcription.

### Evidence that Gro function in the wing disc requires histone deacetylase activity

We also found that histone deacetylase activity is required for Gro function in vivo. Specifically, we found that wing defects resulting from Gro overexpression are significantly attenuated when histone deacetylase activity is diminished, either by treatment of larvae with histone deacetylase inhibitors or by reduction of *Rpd3* gene dosage.

The breadth and severity of wing patterning defects associated with Gro overexpression demonstrate the pleiotropic nature of Gro in development and the sensitivity of the gene regulatory network to the concentration of functional Gro in the nucleus. This concentration sensitivity is not necessarily expected. This is because studies of Gro targets have tended to focus on the mechanisms that modulate the concentration or activity of the repressors that recruit Gro rather than on the mechanisms that modulate the concentration or activity of Gro itself. For example, Brinker and Dorsal, two Gro-dependent repressors, are both distributed in nuclear concentration gradients where they repress target genes in a concentration-dependent manner [41], [42]. The naïve view has been that Gro is simply a component of the constitutive repression machinery that must be present to interpret such repressor concentration gradients. Countering this idea, however, a number of studies suggest that repression of Gro targets is exquisitely sensitive to the level of Gro activity, and indeed one very important way to regulate repression seems to be the modulation of Gro activity levels through Gro phosphorylation [43], [44], [45].

Gro is a critical player in many of the pathways that regulate wing development including the Notch, Ras, and Dpp pathways [45], [46]. Considering these multiple roles, the significant rescue of wing patterning defects by histone deacetylase inhibitors suggests that histone deacetylase-dependent mechanisms of Gro-dependent repression must play important roles in wing development. However, it is also important to note that the histone deacetylase inhibitors do not, on their own, result in wing patterning defects at the concentrations used in these studies (data not shown). Furthermore, although reduction of the Rpd3 gene dosage reduces Gro overexpression phenotypes, otherwise wild-type flies with one copy of *Rpd3* show no developmental defects (data not shown). These observations are likely explained by the existence of redundant mechanisms for repression. As noted above, Gro probably functions via multiple repression mechanisms. Moreover, many repressors, including Brinker, Hairy and Knirps, that interact with Gro also interact with other corepressors such as dCtBP [15], [17], [30]. Gro and these additional corepressors may function through histone deacetylase-independent mechanisms.

In addition to the role of histone deacetylase in regulating the wing patterning defects that arise from Gro overexpression, we specifically demonstrated a role for histone deacetylase in repression via a defined cis-regulatory module, the *vgQ* enhancer [33]. As with the wing patterning defects, the ability of Gro to direct ectopic repression decreases in a dose dependent manner as the concentration of histone deacetylase inhibitor is increased. Proper regulation by this cis-regulatory module is important for wing disc patterning and proliferation, lending further support to the notion that histone deacetylase-dependent Gro-mediated repression plays an important role in wing development.

### Groucho-mediated repression by histone deacetylase-dependent increased nucleosome density

Finally, we examined the changes in chromatin structure at the *vgQ* reporter gene directed by Gro and we explored the role of histone deacetylation in mediating these changes. As demonstrated here, overexpression of Gro results in increased repression of the *vgQ* reporter gene in the wing disc. This increased repression is accompanied by an increase in the nucleosome density in the region flanking the site of Brinker-mediated Gro recruitment. A related observation of increased nucleosome density has been observed for a reporter gene repressed by Hairy-mediated Gro recruitment in the *Drosophila* embryo [27]. In this study, increased nucleosome density was observed at the Gro recruitment site, but not at more distal regions. This may reflect functional differences in the regulatory factors associated with the different cis-regulatory elements, or it could reflect differences in mechanisms of Gro mediated repression in the embryo versus the wing disc. More recently, Moshkin et al. demonstrated that RNAi-mediated knockdown of the transcription factor Hairless and the histone chaperone NAP1 leads to increased nucleosome density at *Enhancer of split* promoter and enhancer regions [47]. It is possible that this change in nucleosome density requires Gro, since it has been shown to associate with the Suppressor of Hairless/Hairless complex [48]. Although these data suggest that Gro-mediated repression is associated with increased nucleosome density, we cannot, at this point, conclude that the increase in nucleosome density is essential for repression or is the only mechanism by which Gro represses transcription. For example, a recent study in yeast demonstrated that the repressor Gal80, which mediates an increase in nucleosome density, can repress endogenous target genes under conditions where the nucleosome density increase is prevented [49]. In addition, since we have not examined target genes or reporters known to lack binding sites for repressors that recruit Gro, we cannot definitively conclude that the increase in nucleosome density is due to gene specific recruitment of Gro and Rpd3. It is formally possible that Gro mediates global histone deacetylase-dependent changes in nucleosome density that may be required, but not sufficient for repression.

Gro-mediated histone deacetylation could lead directly to increased nucleosome density by stabilizing histone/DNA or internucleosomal interactions [50], [51], [52]. This possibility is supported by the finding that TSA treatment, which leads to histone hyperacetylation, leads to a marked decrease in nucleosome density at the *vgQ*-lacZ reporter. Since Gro binds to deacetylated nucleosomes [22], the increased density of deacetylated nucleosomes might in turn be expected to increase Gro density at the target gene. Contrary to this idea, we observed no change in Gro density at the reporter when histone deacetylase is inhibited with TSA. This suggests that the interaction between Gro and the target gene is stabilized by more than a binary interaction between Gro and hypoacetylated histone tails. Groucho family proteins function within a corepressor complex containing other chromatin binding proteins such as Sin3, HDAC1, and RbAp48 [25]. It is possible that multiple members of this corepressor complex contribute to association of Gro with chromatin.

While interactions between Gro and deacetylated histone tails may not be required to maintain association of Gro with a target gene, they could nonetheless play an important role in modulating chromatin structure and/or nucleosome density. This idea is consistent with *in vitro* studies showing that the Gro homolog Grg3 binds nucleosomal arrays in a histone-tail-dependent manner to create nucleosomal aggregates [26].

In conclusion, these findings support a model in which Gro directs the formation of a local chromatin environment characterized by high density, deacetylated nucleosomes that may be inaccessible to the transcriptional machinery. The formation of this region of high nucleosome density relies on histone hypoacetylation. Future experiments will address the relationship between Gro-mediated changes in chromatin structure, transcription machinery recruitment, and elongation.

## Materials and Methods

### Cell culture, transfection and RNAi

*Drosophila* S2 cells were cultured at 24°C in Schneider's insect medium (Sigma) supplemented with 10% heat-inactivated fetal bovine serum. Transfections were performed in triplicate on cells growing in 6-well plates, using the calcium phosphate method. The luciferase reporter G_5_DE_5_tkLuc (10 µg), the internal control reporter pRL-TK (1 µg) (Promega), pPac-Dorsal (0.12 µg), pPac-Twist (0.4 µg) and pPac-Gro (30, 100 or 300 ng) constructs were cotransfected with 1 µg of dsRNA for either Dorsal, Gro or Rpd3. The reporter gene expression was quantified after 48 h using the Dual Luciferase Reporter System (Promega). dsRNA was prepared by creating linear templates by PCR with primers containing flanking T7 promoters. The linear templates were gel purified (Qiagen) and used for *in vitro* transcription (Megascript). Primers for Dorsal dsRNA are 5′-TAATACGACTCACTATAGGGAGCGAGCAACTACAACCACAACA-3′ and 5′-TAATACGACTCACTATAGGGAGTTACGTGGATATGGACAGGTT-3′. Primers for Gro dsRNA are 5′-TAATACGACTCACTATAGGGAGATGTATCCCTCACCGGTGC-3′ and 5′-TAATACGACTCACTATAGGGAGTGAGTGGGATTCCATTTCATT-3′. Primers for Rpd3 dsRNA are 5′-TAATACGACTCACTATAGGGAGCTGGAGAAGATCAAGAACCGT-3′ and 5′-TAATACGACTCACTATAGGGAGAATGTTGTTCTCCTTGGCG-3′. For qRT-PCR, total RNA was isolated from cells with Trizol (Invitrogen) and treated with DNase. cDNA synthesis was performed with M-MLV Reverse Transcriptase (Invitrogen) and 5 µg of RNA. qPCR was performed with FastStart SYBR Green Master mix (Roche) and Rpd3 levels were normalized to Actin5c. All constructs have been previously published [23].

### Cell culture chromatin immunoprecipitation assays

Stable *Drosophila* cell lines containing pRM-GAL4Gro and G_5_DE_5_tkLuc were treated 500 µM CuSO_4_ to induce GAL4Gro expression. Forty-eight hours later, cells were fixed with 1% formaldehyde for 1 hour and sonicated to result in an average DNA size of 500 bp. Chromatin was immunoprecipitated with the following polyclonal antibodies: Gro, Rpd3, acetyl-Histone H3 (Lys 9) (Millipore #06-942), acetyl-Histone H3 (Lys 14) (Millipore #06-911), acetyl-Histone H4 (Lys 5) (Millipore #06-759), acetyl-Histone H4 (Lys 12) (Millipore #06-761), acetyl-Histone H4 (Lys 16) (Millipore #06-762), acetyl-Histone H4 (Lys 8) (Millipore #06-760) and Anti-Histone H3 (Abcam #ab1791). Immunoprecipitated DNA was analyzed by qPCR using the FastStart SYBR Green Master mix (Roche). For a more detailed protocol including buffers, see Millipore's online ChIP protocol. The ChIP signal was quantified as a percentage of the input. More specifically, 10% of the ChIP input was set aside and purified along with the immunoprecipitated DNA. Five 3-fold serial dilutions of the input were prepared and quantified by qPCR along with the immunoprecipitated DNA. A standard curve was plotted with the quantities of the diluted input (% input vs. c(t)). Linear regression analysis was then used to quantify the immunoprecipitated DNA.

### *Drosophila* wing disc chromatin immunoprecipitation assays

Wing discs were dissected from 3^rd^ instar larvae in Shields and Sang M3 Insect Medium (Sigma) without serum. Discs were fixed with 3% formaldehyde for 30 minutes. Discs were lysed by multiple liquid nitrogen freeze/thaw cycles and mashing with a pipette tip. Discs were biorupted to an average DNA fragment size of 500 bp. Chromatin was immunoprecipitated with the following polyclonal antibodies: Gro, Anti-aceytl-Histone H3 (Millipore #06-599), Anti-acetyl-Histone H4 (Millipore #06-866), and Anti-Histone H3 (Abcam #ab1791). Immunoprecipitated DNA was analyzed by qPCR using the FastStart SYBR Green Master mix (Roche). The ChIP signal was quanitifed as described above for the cell culture chromatin immunoprecipitation assays.

### HDAC inhibitor assays

HDAC inhibitor food was prepared by mixing 0.93 g agar, 6.12 g cornmeal, 12.94 g dextrose, 3.24 g dry yeast and water up to 100 ml. Ingredients were heated to 80°C and mixed on a stir plate until a fluid consistency was reached. The mixture was cooled and just prior to pouring into vials, 1 ml of 10% methylparaben (in EtOH), HDAC inhibitor (in DMSO), and food coloring were added. After food coloring showed good mixing, food was poured into vials and stored at 4°C until use.

### Adult wing preparation

Adult wings were dissected and placed in 100% MetOH for a few minutes for fixation. Wings were then equilibrated in a solution of 60% Glycerol/40% PBS and directly mounted on glass slides. Wings were imaged with a Zeiss Axioskop microscope (4X objective, brightfield).

### Wing disc immunostaining

Third instar larvae were inverted in PBS and fixed in 4% formaldehyde for 40 minutes. They were washed in PBST, blocked in 10% bovine calf serum (BCS), and incubated with primary antibody overnight at 4°C (rabbit anti-β-galactosidase, 1∶10000, ICN) (mouse anti-Gro, 1∶100, kind gift from Christos Delidakis). Larvae were again washed in PBST, blocked in 10% BCS, and incubated in secondary antibody for 2 hrs at room temperature (goat anti-mouse 488 nm and goat anti-rabbit 568 nm, 1∶1000, Invitrogen). Larvae were then stained in PBST w/300 nM DAPI and washed in PBST alone. Wing discs were dissected and imaged on a Deltavision microscope.

### Drosophila lines

*UAS-Gro^WT^* flies have been previously described [30]. Expression was driven with the *C765-GAL4*, *MS1096-GAL4* (Bloomington #8696) and *Ser-GAL4* (Bloomington #6791) drivers. *Rpd3^Δ^* was created by an imprecise excision of the P-element in the hypomorphic *Rpd3^04556^* allele resulting in ∼2000 bp deletion that removed the 1^st^, 2^nd^ and part of the 3^rd^ exon.

## Supporting Information

Figure S1 Trichostatin A and HC-Toxin rescue wing vein patterning defects resulting from MS1096-Gal4 driven Gro overexpression at 18°C. Overexpression of Gro directed by the 3rd instar wing disc driver MS1096-Gal4 in females at 18°C leads to PCV, L4 and L5 vein patterning defects (B-D). Flies raised in TSA and HC-Toxin show a decrease in vein patterning defects (A and data not shown). A dose dependent decrease in PCV (E), L4 (F), and L5 (G) defects are observed with both TSA (diamonds) and HC-Toxin (squares).
(2.12 MB TIF)

Figure S2 Gro-mediated increase in nucleosome density within the vestigial quadrant enhancer in the eye-antennal disc. Overexpression of Gro with the GMR-Gal4 driver at 29°C results in a significant increase in eye-antennal disc histone H3 ChIP signal (green bars) in areas flanking the Gro recruitment site in the vgQ-lacZ transgene when compared to flies not overexpressing Gro (orange bars).
(0.10 MB TIF)

Table S1 Statistical analysis of data in Figure S1. P-values of wing phenotypes calculated by Fisher's exact test and number of wings sampled.
(0.38 MB TIF)

Table S2 Statistical analysis of data in Figure 3. P-values of wing phenotypes calculated by Fisher's exact test and number of wings sampled.
(0.26 MB TIF)
