# Supplementary figures and images for: Groucho-Mediated Repression May Result from a Histone Deacetylase-Dependent Increase in Nucleosome Density
Source: PLoS One. 2010 Apr 13;5(4):e10166. doi: 10.1371/journal.pone.0010166 (PMC2854148; doi:10.1371/journal.pone.0010166)

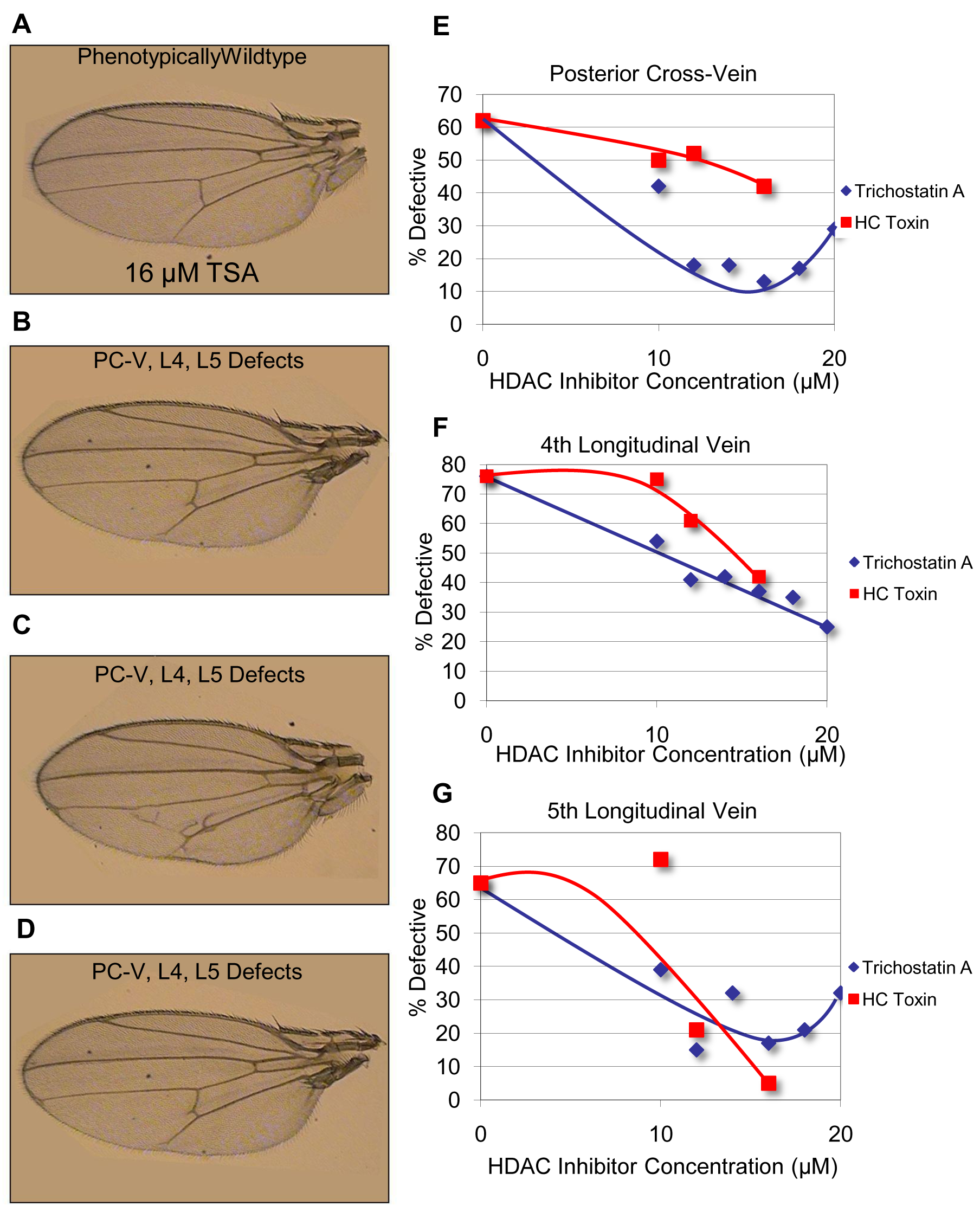

Supplement: Figure S1 — Trichostatin A and HC-Toxin rescue wing vein patterning defects resulting from MS1096-Gal4 driven Gro overexpression at 18°C. Overexpression of Gro directed by the 3rd instar wing disc driver MS1096-Gal4 in females at 18°C leads to PCV, L4 and L5 vein patterning defects (B-D). Flies raised in TSA and HC-Toxin show a decrease in vein patterning defects (A and data not shown). A dose dependent decrease in PCV (E), L4 (F), and L5 (G) defects are observed with both TSA (diamonds) and HC-Toxin (squares). (2.12 MB TIF) [file pone.0010166.s001.tif]

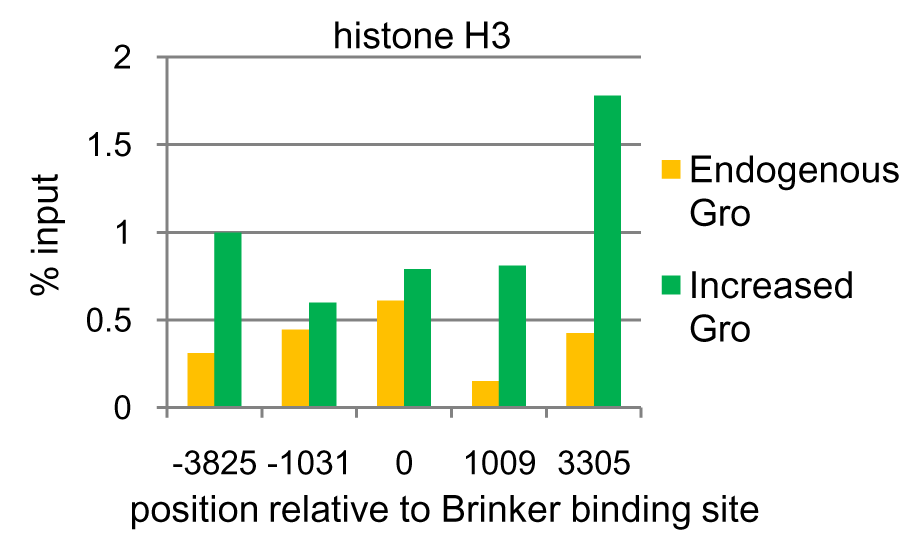

Supplement: Figure S2 — Gro-mediated increase in nucleosome density within the vestigial quadrant enhancer in the eye-antennal disc. Overexpression of Gro with the GMR-Gal4 driver at 29°C results in a significant increase in eye-antennal disc histone H3 ChIP signal (green bars) in areas flanking the Gro recruitment site in the vgQ-lacZ transgene when compared to flies not overexpressing Gro (orange bars). (0.10 MB TIF) [file pone.0010166.s002.tif]

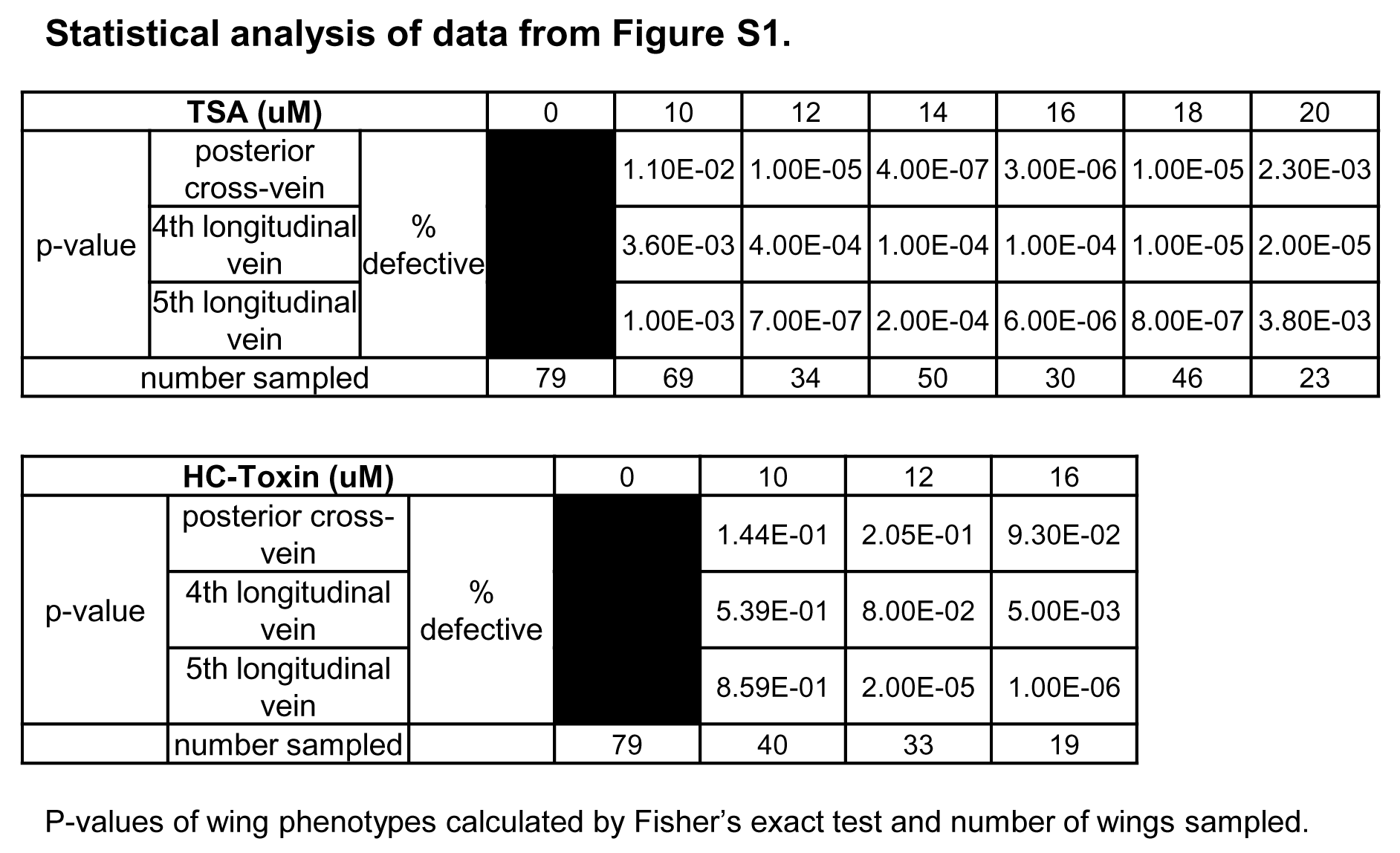

Supplement: Table S1 — Statistical analysis of data in Figure S1. P-values of wing phenotypes calculated by Fisher's exact test and number of wings sampled. (0.38 MB TIF) [file pone.0010166.s003.tif]

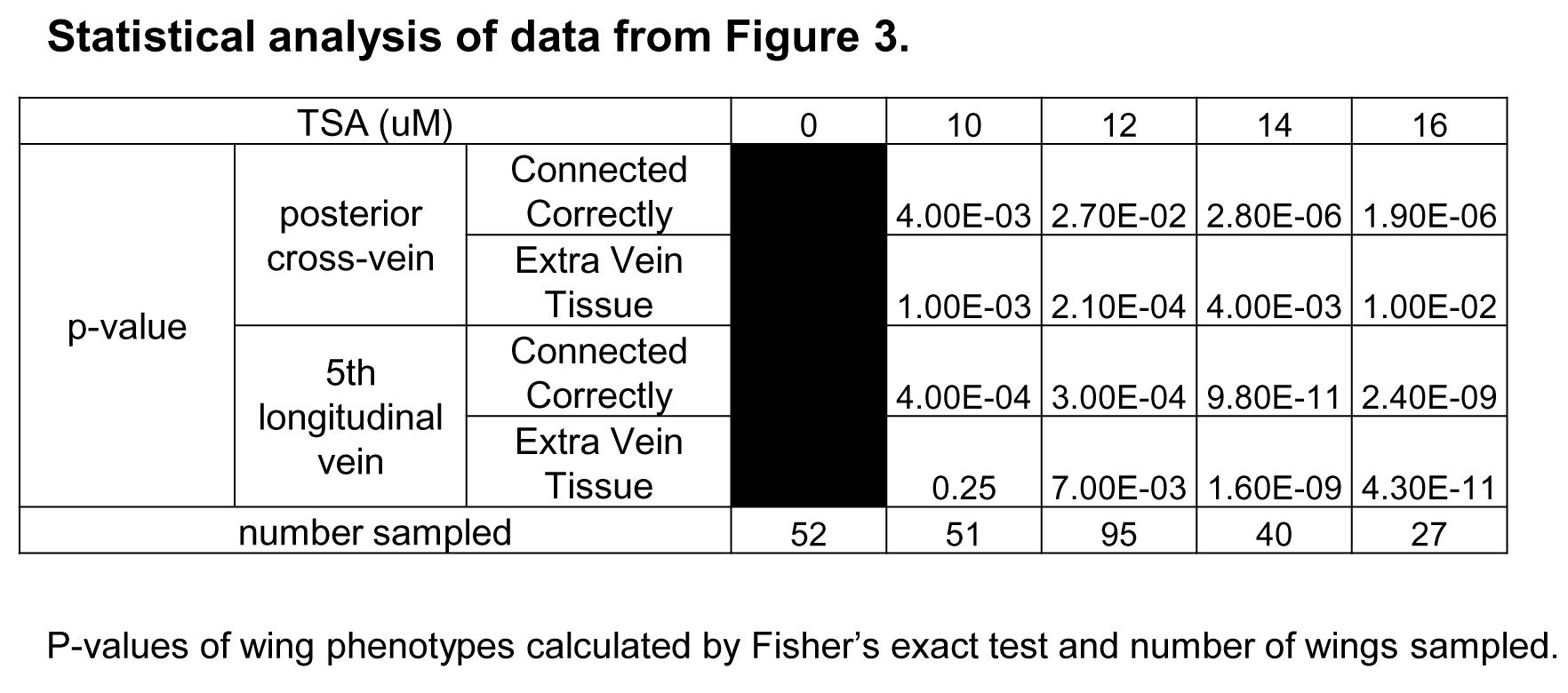

Supplement: Table S2 — Statistical analysis of data in Figure 3. P-values of wing phenotypes calculated by Fisher's exact test and number of wings sampled. (0.26 MB TIF) [file pone.0010166.s004.tif]
